# Structural Magnetic Resonance Imaging-Based Surface Morphometry Analysis of Pediatric Down Syndrome

**DOI:** 10.3390/biology13080575

**Published:** 2024-07-30

**Authors:** Jacob Levman, Bernadette McCann, Nicole Baumer, Melanie Y. Lam, Tadashi Shiohama, Liam Cogger, Allissa MacDonald, Emi Takahashi

**Affiliations:** 1 Department of Computer Science, St. Francis Xavier University, Antigonish, NS B2G 2W5, Canada; 2 Athinoula A. Martinos Center for Biomedical Imaging, Massachusetts General Hospital, Harvard Medical School, Charlestown, Boston, MA 02129, USA; 3 Nova Scotia Health Authority, Halifax, NS B3H 1V8, Canada; 4 Department of Human Kinetics, St. Francis Xavier University, Antigonish, NS B2G 2W5, Canadamlam@stfx.ca (M.Y.L.); 5 Department of Neurology, Boston Children’s Hospital, 300 Longwood Ave, Boston, MA 02115, USA; 6 Department of Pediatrics, Graduate School of Medicine, Chiba University, Chiba 260-8677, Japan; 7 Department of Education, St. Francis Xavier University, Antigonish, NS B2G 2W5, Canada; 8 Department of Biology, St. Francis Xavier University, Antigonish, NS B2G 2W5, Canada; 9 Division of Newborn Medicine, Department of Medicine, Boston Children’s Hospital, Harvard Medical School, 401 Park Dr., Boston, MA 02215, USA; 10 Department of Radiology, Harvard Medical School, Boston, MA 02115, USA

**Keywords:** down syndrome, surface morphometry, magnetic resonance imaging

## Abstract

**Simple Summary:**

Down syndrome (DS) is a genetic disorder caused by an additional partial or full copy of chromosome 21. Analysis of the brain’s surface can potentially assist in providing a better understanding of structural brain differences, and may help characterize DS-specific brain development. We performed a study of 73 magnetic resonance imaging (MRI) examinations of DS patients (aged 1 day to 22 years) and compared them to a large cohort of 993 brain MRI examinations of neurotypical participants, aged 1 day to 32 years. A variety of measurements that characterize the surface of the brain were extracted from each brain region in each examination. Results demonstrate broad reductions in surface area and abnormalities of surface curvature measurements across the brain in DS. Findings suggest the presence of developmental abnormalities of the brain’s surface in DS that can be characterized from clinical MRI examinations.

**Abstract:**

Down syndrome (DS) is a genetic disorder characterized by intellectual disability whose etiology includes an additional partial or full copy of chromosome 21. Brain surface morphometry analyses can potentially assist in providing a better understanding of structural brain differences, and may help characterize DS-specific neurodevelopment. We performed a retrospective surface morphometry study of 73 magnetic resonance imaging (MRI) examinations of DS patients (aged 1 day to 22 years) and compared them to a large cohort of 993 brain MRI examinations of neurotypical participants, aged 1 day to 32 years. Surface curvature measurements, absolute surface area measurements, and surface areas as a percentage of total brain surface area (%TBSA) were extracted from each brain region in each examination. Results demonstrate broad reductions in surface area and abnormalities of surface curvature measurements across the brain in DS. After adjusting our regional surface area measurements as %TBSA, abnormally increased presentation in DS relative to neurotypical controls was observed in the left precentral, bilateral entorhinal, left parahippocampal, and bilateral perirhinal cortices, as well as Brodmann’s area 44 (left), and the right temporal pole. Findings suggest the presence of developmental abnormalities of regional %TBSA in DS that can be characterized from clinical MRI examinations.

## 1. Introduction

### 1.1. Down Syndrome

Down syndrome (DS) is a common chromosomal disorder [1], with an estimated prevalence of 12.6 per 10,000 in the United States [2]. The overexpression of chromosome 21 genes leads to both cognitive and physical developmental abnormalities [3]. Cognitive deficits include reductions in memory [4,5,6], deficits in learning [7,8], and challenges with language and speech [9,10]. Children with DS are often delayed in both fine and gross motor skill development, which may be attributed to delayed myelination, hypotonia, and ligamentous laxity [11]. In addition, people with DS are at increased risk of congenital cardiac disease, hearing loss, psychiatric conditions, ophthalmological disorders, and Alzheimer’s disease (AD) [12,13,14].

Despite the risk of additional health conditions in people with DS, expected lifespan and quality of life have increased [15]. A literature review revealed that in 1929, DS life expectancy was 9 years, whereas a systematic review conducted in 2010 indicated that DS patients are expected to live approximately 60 years [16]. In developed countries, such as Australia, improvements in life expectancy have been observed from 18 to 60 years from 1963 to 2002 [17], observations that parallel the life expectancy gains observed in America [1]. This prolonged lifespan could be attributed to enhancements in the standard of patient care [16], including improved prognosis through earlier treatments of respiratory infections and heart disease [18], and potential improvements from a shift from institutional care to community living [19]. Research characterizing DS’s effect on brain development may yield more effective treatments and interventions, by identifying brain regions that can be targeted for therapies, and potentially acting as treatment monitoring technologies, which could contribute to future gains in expected lifespan.

### 1.2. Magnetic Resonance Imaging and Associated Analytic Software

Neuroimaging has been employed to characterize phenotypic abnormalities towards better understanding brain development in DS. Magnetic resonance imaging (MRI) creates physiological and anatomical images of a patient. Investigating individuals with physical and cognitive abnormalities with MRI can identify regional brain structures and potentially associated developmental, behavioral, and neurological symptoms. Automated brain MRI analytics software [20] produces regionally distributed surface area measurements across the brain by exploiting the contrast between gray matter (GM), white matter (WM), and cerebrospinal fluid (CSF).

### 1.3. Literature

Past research studies have focused on characterizing the brains of participants with DS via MRI; however, they have generally focused on regional volumetrics and have reported pervasive deficits in both total and regional brain volumes in DS [21,22,23,24,25,26,27,28,29,30,31,32,33,34,35,36,37,38,39,40]. More recently, these findings have been expanded upon to fetal analyses, which have reported fetal cortical and cerebellar developmental abnormalities [41,42]. Such volumetric approaches to analyzing MRI brain scans are more common than surface-based analyses, which have the potential to improve characterization of cortical development beyond that which can be accomplished by volumetric analyses alone. Studies that have used this latter approach have shown a reduction in surface area (primarily frontal and temporal regions) in DS youth [43]. More recent studies have moved beyond examining children and adults and investigated the developing brain with fetal and neonatal imaging. For example, one study compared various brain measures at varying time points of gestation and noted a significantly smaller vermis surface area in DS in the second trimester [42]. Another study revealed that the fetal brains of those with DS had “smaller growth trajectories of the inner cerebral surface area” compared to the fetal brains of typically developing controls [41]. Finally, a stereological study undertaken by Karlsen and Pakkenberg [44] examined cortical surface area in female participants with DS (ages 61 to 70 years), and reported considerable decreases in surface areas relative to a group of female controls (ages 60 to 80 years) in the frontal, temporal, and parietal regions, but the occipital region, which also exhibited lowered average surface area in DS, was not a statistically significant finding [44], in a poorly statistically powered study with just four patients with DS and six controls.

Here, we hypothesize that regional surface morphometry measurements, inclusive of surface curvature measurements and characterizing the brain’s regional surface area as a percentage of total brain surface area, may help characterize abnormal neurodevelopment associated with DS. Surface morphometry measurements have the potential to assist in characterizing topology, which in the cerebral cortex presents as a highly convoluted two-dimensional sheet [45]. Traditional volumetric analyses [31] do not address the topological presentation of the brain’s cortex. Since cortical folding and sulci formation heavily affect the topology of the brain, and since they have been implicated as potentially important developmental characteristics of the brain [46,47], analyzing the DS population as part of a surface morphometry analysis may assist in better characterizing the presentation of the brain in DS with biomarker measurements that are more sensitive to cortical folding and sulci formation than traditional biomarkers, such as regional volumes. We expect previous literature findings, outlined above, of reduced absolute regional surface areas, to be confirmed in our analysis, and we expect to characterize additional regional brain abnormalities with surface area measurements considered in this study but not included in previous analyses, such as surface curvature, and characterizing regional surface area as a percentage of total brain surface area.

## 2. Materials and Methods

### 2.1. Participants

We investigated the presentation of the brain from MRI examinations obtained as part of a large-scale retrospective analysis, from which the participants included were previously analyzed as part of two earlier studies on DS, focused on the thickness of the cortex [48] and volumetrics [31]. This manuscript is focused on surface morphometry-based regional biomarkers.

“Following approval by Boston Children’s Hospital’s (BCH) Institutional Review Board (who waived the need of informed consent due to lack of risk to participants in this retrospective analysis), the BCH clinical imaging electronic database was reviewed from 1 January 2008 until 24 February 2016, and all brain MRI examinations of participants aged 0 to 32 years were included for further analysis if DS was indicated in the participant’s electronic medical records. Examinations deemed low quality (due to excessive participant motion, large metal artifact from dental hardware, lack of a T1 structural imaging volume providing diagnostically useful axial, sagittal and coronal oriented images, etc.) were excluded from this analysis. Examinations that were inaccessible due to technical reasons were also excluded. This generated a total of 73 examinations from DS participants. 73% of our DS examinations included patients with congenital heart defects, according to their medical records. The neurotypical cohort was assembled retrospectively in a previous analysis (Levman et al., 2017), where participants were selected on the basis of normal MRI examinations, as assessed by a BCH neuroradiologist, and medical records with no indication of any neurological problems (participants with a known disorder such as autism, cerebral palsy, traumatic brain injury, developmental delay, tuberous sclerosis complex, stroke, neurofibromatosis, epilepsy, attention deficit hyperactivity disorder, etc. were excluded). Participants with cancer were also excluded in order to avoid data exhibiting growth trajectories that are affected by treatments such as chemotherapy. The exclusion criteria used for the DS participants was also applied to the neurotypical participants yielding 993 examinations. Demographic information on studied participants is presented in Table 1, the information therein having been previously presented (Levman et al., 2019)” [31].

### 2.2. MRI Data Acquisition and Preprocessing

The acquisition and preprocessing of the data included in this analysis was previously completed as part of earlier analyses on DS, focused on the thickness of the cortex [48] and a volumetric analysis [31].

“All participants (both DS and neurotypical) were imaged with clinical 3 T MRI scanners (Skyra, Simens Medical Systems, Erlangen, Germany) at BCH yielding T1 structural volumetric imaging examinations which were accessed through the Children’s Research and Integration System (Pienaar et al., 2014). There is variability in the pulse sequences employed to acquire these volumetric T1 examinations due to the clinical and retrospective nature of this study, with spatial resolution in the x and y directions varying from 0.2 to 1.4 mm (0.9 mm on average) and through plane thickness varying from 0.5 to 2 mm (1 mm on average). Strengths and limitations of the large-scale varying MR protocol approach used in this study are addressed in the Discussion. A single volumetric MRI was acquired from each imaging session, with some patients returning for multiple MRI examinations (different imaging sessions) which were used in the analysis. Motion correction was not performed, but examinations were visually assessed and those with substantial motion artifacts were excluded. T1 structural examinations were processed with FreeSurfer (Fischl, 2012), using the recon-all command to align the input examination to all available brain atlases. Those atlases that include volumetric measurements were included for further analysis (atlases: aseg, aparc, aparc.a2009s, aparc.DKTatlas40, BA, BA.thresh, entorhinal_exvivo, wmparc). These combined atlases include definitions of 232 brain regions from which volumetric measurements were extracted. Each FreeSurfer output T1 structural examination was displayed with label map overlays and was visually examined for quality of regional segmentation results. Exams were excluded from this analysis if FreeSurfer results were observed to substantially fail (i.e., FreeSurfer regions-of-interest (ROIs) that did not align to the MRI and examinations where major problems were observed with an ROI such as a cerebellar segmentation extending far beyond the extent of the cerebellum).

In our DS cohort, these criteria resulted in the exclusion of 1 exam due to a segmentation error, 36 due to technical problems accessing examinations, 65 due to lack of available volumetric examination (thus being incompatible with FreeSurfer technology), 1 due to no non-contrast enhanced volumetric exam, 1 due to a motion artifact and 31 due to FreeSurfer’s failure to complete execution on the patient’s exam. Thus, our final inclusion of 73 examinations represents 35% of all DS MRI examinations available. In our healthy cohort, 58 exams were excluded due to FreeSurfer’s failure to complete execution on the patient’s exam, 1 due to major motion artifact, 1 due to an imaging artifact, 231 due to lack of volumetric examination, 7 due to no non-contrast enhanced volumetric exam and 20 due to technical problems accessing the examinations. The DS group had considerably higher rates of exclusions, which is likely related to the additional challenges in successfully imaging this cohort. The overall rates of motion artifacts are low in both groups, because at BCH, the MR technicians repeat an additional structural MRI examination when motion artifacts are observed. Thus, imaging sessions produce 1–3 volumetric examinations per patient, one of which was selected for this study based on imaging quality” [31].

### 2.3. Statistical Analysis

For each cortical region under consideration, biomarkers acquired include the absolute surface area, the folding index, the intrinsic curvature index (unitless), the integrated rectified mean curvature, and the integrated rectified Gaussian curvature. The regional cortical surface areas have units of (mm^2^). The folding index is a single number summarizing the overall amount of folding on a cortical surface and is unitless. The intrinsic curvature index is a “natural” index, which should be 1 for FreeSurfer surfaces and is unitless. Mean curvature is calculated as the mean (or average) of the two principal curvatures (units mm^−1^), whereas Gaussian curvature is the product (multiplication) of the two principal curvatures (units mm^−2^). Higher curvature values imply that the folding of the brain is “sharper” in at least one direction.

To analyze data based on age, participants were categorized into four groups: early childhood (ages 0 to <5 years), late childhood (ages 5 to <10 years), early adolescence (ages 10 to <15 years), and late adolescence (ages 15 to 20 years). Since there were few participants ages 20 years or older, they were not included in the group-wise analyses but are provided in the scatter plots presented for completeness. It should also be noted that the sample size is small in the 15–20 year age group (*n* = 9), making that component an exploratory analysis. All absolute regional surface area measurements were assessed using group-wise comparisons and reassessed as a percentage of total brain surface area (%TBSA). The %TBSA was calculated as the regional surface area measurement (in mm^2^) normalized by the total brain surface area (also in mm^2^) as assessed by combining the left and right hemisphere’s estimated surface areas (lh.curv.stats Raw Total Surface Area + rh.curv.stats Raw Total Surface Area), respectively [20]. This resulted in a total of 2180 regionally distributed biomarker measurements included for analysis per age group.

The group-wise comparisons for the acquired measurements were assessed for each age group using Cohen’s d statistic. Positive/negative values indicated a higher/lower mean value, respectively, in DS relative to neurotypicals. The d statistic was reported as it is an established effect size assessment method. For each biomarker from each brain region compared, a *t*-test-based *p*-value [49] was reported for each age group, yielding *m* = 8720 group-wise comparisons which resulted in Bonferroni-corrected statistical significance of *p* < 0.05/m = 5.73 × 10^−6^.

Finally, a multivariable regression-based statistical model was created (MATLAB R2018a, MathWorks Inc., Natick, MA, USA), adjusting each measurement in each age group to control for differences in both age and gender. Age was treated as a continuous variable and gender as a binary variable for each age group. This model was used on each surface biomarker to assess whether the results observed are the result of naturally occurring age or gender effects.

## 3. Results

**Figure 1:**
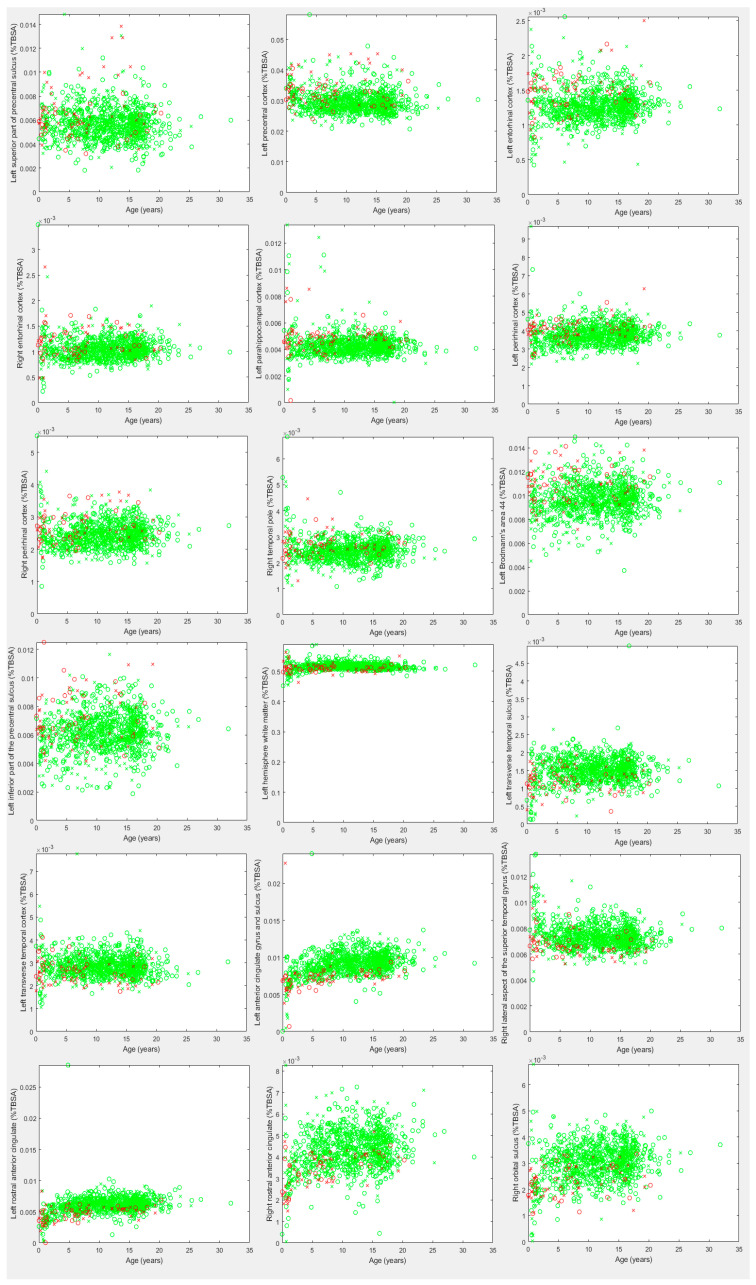
Scatter plots of the leading findings presented in Table 4 and Table 5, representing surface area abnormalities identified in DS as a percentage of total brain surface area (%TBSA). Green samples represent neurotypical participants, red samples represent DS participants. X represents a male, O a female.

The results revealed that several brain regions exceeded the Bonferroni adjusted threshold for statistically significant findings for surface morphometry measurements between the DS and neurotypical groups. Of the 8720 group-wise comparisons conducted, 3.5% were Bonferroni-corrected statistically significant. In the age-dependent analysis using Cohen’s d statistic, we observed a collection of biomarkers that potentially can assist to characterize the brain in DS. Table 2, Table 3, Table 4 and Table 5 present the most prominent biomarkers observed, which are presented in descending order of Cohen’s d statistic. The unadjusted d statistic was reported for ease of interpretation and comparison with future research. When the value met or exceeded the Bonferroni-corrected threshold in at least one age group and on at least one corresponding regional brain biomarker, the corresponding entry was bolded. Figure 1 provides scatter plots for all the findings in Table 4 and Table 5 and a black and white version is available in the Supplementary Materials.

The age-dependent analyses of leading absolute surface area measurements are summarized in Table 2. Almost all significant differences except for one (superior part of the precentral sulcus) were observed to be decreased in DS participants. The majority of the differences across groups were found in age ranges 5 to 10 and 10 to 15 years, in various brain regions through all lobes. There were some regions that started showing abnormalities in DS in ages 0 to 5 years, such as the right transverse temporal gyrus, right lateral aspect of the superior temporal gyrus, bilateral postcentral gyrus, right supramarginal gyrus, left Brodmann’s area 3b, right superior parietal, right Brodmann’s area 2, and the bilateral Brodmann’s area 1. All these regions with early abnormalities, except for the bilateral Brodmann’s area 1, continued to be found in at least either ages 5 to 10 or 10 to 15 years, but never in ages 15–20 years. In the ages of 15 to 20 years, only a few regions such as the right inferior segment of the circular sulcus of the insula, left lateral orbitofrontal gyrus, left orbital gyrus, left medial orbital sulcus, and right middle-anterior cingulate gyrus and sulcus were found to show abnormalities across groups. All of them were also found to be abnormal in ages 5 to 10 years, but not in other age ranges. Given the small number of regions with abnormalities found in ages 15 to 20 years, it is notable that the majority of such regions were located in/around the orbitofrontal area.

Table 3 shows leading surface curvature measurements in age-dependent analyses. Most of the differences were found in ages 15 to 20 years. The intrinsic curvature indices of the whole brain curvatures and bilateral whole hemisphere folding indices were decreased in DS in ages 0 to 5, 5 to 10, and 10 to 15 years, but not in ages 15 to 20 years. There are some other measurements that showed abnormalities in ages 5 to 10 and 10 to 15 years, such as mean curvatures of the left medial occipito-temporal/lingual sulci, left lingual gyrus, left cuneus gyrus, V1 and V2 in ages 5 to 10 years, and those of the left transverse frontopolar gyri and sulci, bilateral rostral middle frontal, right pars orbitalis, right Brodmann’s area 45, left orbital gyrus, and Gaussian curvatures of the left medial occipito-temporal/lingual sulci in ages 5 to 10 years and those of the right pericallosal sulcus and right posterior cingulate in ages 10 to 15 years, but interestingly, none of them continued to show the abnormalities in ages 15 to 20 years. Unfortunately, our 15 to 20-year age cohort is poorly statistically powered, so forming firm conclusions from this portion of the analysis is challenging.

Leading surface area increases as a % of TBSA are summarized in Table 4, and leading surface area decreases as a % of TBSA are summarized in Table 5. The majority of the leading measurements were found in ages 10 to 15 years. Although the increase in the surface area (%TBSA) in the left superior part of the precentral sulcus (Table 4) and the decrease in the surface area (%TBSA) in the left transverse temporal gyrus and sulcus (Table 5) in ages 10 to 15 years were also observed before calculating %TBSA in Table 2, %TBSA exhibited regions with increased surface area mainly in the ventral temporal areas (bilateral entorhinal and perirhinal cortices and left parahippocampal cortex) in ages 10 to 15 years. Interestingly, all the increased or decreased measurements in Table 4 and Table 5 were age-range specific and were not found in the other age ranges.

## 4. Discussion

The current study involved a large-scale retrospective surface morphometry analysis of MRI examinations in a group with DS compared with a neurotypical group. Findings include abnormalities in absolute surface area, curvature/folding, surface area as %TBSA, and the ventral temporal regions, which are outlined below. To the best of our knowledge, this was the first study to consider surface curvature measurements, as well as regional surface area measurements as a percentage of total brain surface area, findings that are outlined in the second and third subsections below. However, we will first compare our results on absolute surface area, with those available in the literature.

### 4.1. Absolute Surface Area

We observed extensive reduced regional absolute surface areas, which is in line with the literature [43]. The majority of the differences across groups were found in age ranges of 5 to 10 and 10 to 15 years, in various brain regions through all lobes. There were only some regions that started showing abnormalities in DS in ages 0 to 5 years, such as the right transverse temporal gyrus, right lateral aspect of the superior temporal gyrus, bilateral postcentral gyrus, right supramarginal gyrus, left Brodmann’s area 3b, right superior parietal, and right Brodmann’s area 2. Abnormal absolute surface areas in these regions could be early biomarkers until 15 years old, because all these regions with early abnormalities continued to be found in at least either ages 5 to 10 or 10 to 15 years, but never in ages 15 to 20 years. We previously showed that Brodmann’s area 3b had large effect sizes associated with increased mean cortical thickness starting from the ages of 0 to 5 years [48] and increased regional volume at 5 to 10 years [31], which could be in line with the current results.

In the 15 to 20-year age group, only a few regions were found to have abnormalities across groups. Given the small number of regions with abnormalities found in this age group, it is notable that most of such regions were located in/around the orbitofrontal area. However, it should be noted that our analyses on the 15 to 20-year old cohort are poorly statistically powered, and so future work on datasets with larger sample sizes are needed to further assess these findings.

Previous research has reported “smaller growth trajectories of the inner cerebral surface area” compared to the fetal brains of typically developing controls [41]. Delayed or reduced growth of the cerebral surface can potentially result in delayed development of cortical folding and sulci formation, as it is known that differential growth of the cortex (faster growth) relative to the white matter (slower growth) is a major contributing factor to the emergence of prominent cortical folds and sulci formation [50,51,52,53,54,55]. Evidence in favor of differential growth rates contributing to characteristic sulcal and gyral patterns of the cortex is strong [51]. In this analysis, a physical model was constructed in which the simulated grey matter expands faster than the underlying simulated white matter, repeatedly and consistently resulting in characteristic prominent sulcal and gyral patterns [51]. In DS, we observe delayed growth of the surface of the cortex, which may be linked with delayed grey matter expansion. Based on the experiment outlined above [51], we should thus expect to observe reduced/delayed cortical folding and sulci formation in DS, potentially due to less extreme differential growth rates of the cortex relative to the white matter in DS. Indeed, previous research is very strongly supportive of this, having observed reduced sulcal depth in DS detectable in living fetuses [56]. Additionally, previous research is supportive of reduced sulcal formation and folding, having observed reduced cortical thickness variability in DS [48], resulting in more regular cortical surfaces. The present study confirms reductions in surface area growth in DS (see Table 2).

### 4.2. Curvature/Folding

The findings revealed group-wise differences in various brain regions such as extensive regional increases in surface curvature (see Table 3). To the best of our knowledge, we are the first to report such findings, which may be related to recent reports of regional alterations in cortical sulcal depth; however, we note that this finding was found in living fetuses [54]. Our results showed that DS brains in general had fewer regions exhibiting abnormal curvatures at a young age (0 to 15 years), but increased curvatures at an older age (15 to 20 years) compared to controls (Table 3), coincidently showing almost no statistically significant differences in surface areas (both absolute values [Table 2] and %TBSA [Table 4 and Table 5]) in that age range. Although primary and secondary sulci are formed at earlier developmental stages [55], tertiary sulci continue to develop in adulthood [57,58], which may contribute to the curvature development in DS in 15 to 20 year olds. In addition, given the premature brain developmental status in DS in general, it is also possible that some primary and secondary sulci could still develop in DS in this age range.

There has been a discussion about how brain surface folding emerges during development [59], and given that the absolute surface area and %TBSA both demonstrate abnormalities mostly in 10 to 15 year olds followed by increased curvatures in 15 to 20 year olds, it is possible that changes related to surface area precede curvature changes in DS. However, since the surface area tends to be decreased in DS, future work might benefit from normalizing cortical folding and curvature measurements with the surface area (i.e., emerging folding could be sharper in small surface areas in DS) and therefore could be over-estimated in this study.

### 4.3. Curvatures and %TBSA

Although we observed extensive reduced regional absolute surface areas, which is in line with the literature [43], remarkably, when analyzing surface area measurements as a percentage of total brain surface area (%TBSA), most brain regions do not exhibit statistically significant differences in these normalized surface areas; however, we do report fewer regions exhibiting statistically significant differences between the group with DS and neurotypical group in terms of both positive effects sizes (i.e., measurements where DS exhibits larger %TBSA than the neurotypical group, see Table 4) and negative effect sizes (i.e., measurements where the DS group exhibits smaller %TBSA than the neurotypical group, see Table 5). Those regions that presented with increased %TBSA in the group with DS relative to the neurotypical group included the perirhinal, entorhinal, parahippocampal, and precentral cortices, as well as the temporal pole, Brodmann’s area 44, and the inferior and superior parts of the precentral sulcus (see Table 4). Those regions that presented with decreased %TBSA in the group with DS compared to the neurotypical group included white matter, the transverse temporal gyrus and sulcus, the anterior cingulate gyrus and sulcus, the lateral aspect of the superior temporal gyrus, the rostral anterior cingulate, and the medial orbital sulcus (see Table 5). Once more, to the best of our knowledge, this study is the first to report regional %TBSA abnormalities in DS, and the first to identify the aforementioned regions (Table 4) as exhibiting a %TBSA effect direction inversion in DS relative to absolute/raw regional surface area measurements (i.e., surface areas are smaller in DS generally, but the regions in Table 4 represent increased surface area as measured by %TBSA). In addition, abnormalities in curvatures (Table 3) and %TBSA (Table 4 and Table 5) were found almost exclusively at specific age ranges, which suggests that regional brain development in DS is actively continuous from birth to early adulthood.

### 4.4. Ventral Temporal Regions

The parahippocampal gyrus was previously reported to have increased absolute volumes and increased volume as a percentage of estimated total intracranial volume (%ETIV) in DS relative to neurotypical controls [31] using the same dataset as the present study. The precentral cortex has been previously reported to exhibit abnormally reduced volumes in DS [31] and was one of many brain regions that exhibited abnormally increased volumes in DS as %ETIV [31]; however, in that volumetric analysis, the precentral region was one of a long list of volumetric regions exhibiting increased volume as %ETIV. Similar to the precentral cortex, abnormal increases in perirhinal and entorhinal volumes as %ETIV have previously been reported [31]. It is worth noting that while volumetric abnormalities of these regions have been previously observed, they represent only one of many abnormal volumetric findings. For example, the precentral cortex represented the region of the brain that had the 29th largest effect size, the entorhinal cortex had the 27th largest effect size, the perirhinal cortex had the 15th largest effect size, and the parahippocampal cortex had the 6th largest effect size with respect to %ETIV [31]. In stark contrast, these four cortical regions represent the leading cortical findings in terms of effect sizes when comparing the group with DS to the neurotypical group with respect to %TBSA. These findings imply that the precentral, entorhinal, perirhinal, and parahippocampal cortices may be major sites of abnormal neurodevelopment in DS. We also report abnormal %TBSA in Brodmann’s area 44, in agreement with previous volumetric findings [31], and we also reported abnormal %TBSA in the temporal pole, which has been previously identified as exhibiting reduced cortical thickness variability [48] and increased volume as %ETIV [31].

Also of interest is that this dataset has been previously subjected to a cortical thickness analysis [48], which reported decreased cortical thickness variability in DS relative to neurotypical controls in the parahippocampal gyrus, as well as no findings of either cortical thickness variability, nor mean cortical thickness abnormalities in DS in the perirhinal, entorhinal, and precentral cortices [48]. The results of the current study imply that surface area as a percentage of total brain surface area (%TBSA) may be a sensitive biomarker to abnormal brain development associated with DS and deserves further investigation. It is also worth pointing out that although reporting regional brain volumes as a percentage of total brain volume is common in the literature [31], reporting regional surface areas normalized by total surface area is extremely rare. Although reporting %TBSA is rare, this approach has been used outside of DS research, where it was demonstrated that almost 80% of the surface area of the neocortex is found in the cerebellum [60], early findings that also imply potential value from surface area biomarkers scaled as a percentage of a larger structure’s surface area. Cortical surfaces have been reported to be larger in more intelligent children who were 10 years old [61], implying that cortical surface measurements may be useful in characterizing brain development, and their findings matched our general findings of reduced absolute cortical surface areas in the DS group, a population recognized as having intellectual disabilities [62].

The reporting of surface morphology measurements for the parahippocampal, entorhinal, perirhinal, and precentral cortices are the first in the DS literature. Our analysis considered four age groups and a general trend was observed, in which the youngest age group exhibited the smallest effect sizes in surface morphometry measurements. This suggests that, in this research, the structural abnormalities observed progress through childhood and adolescence. Thus, it is expected that there will be value in longitudinal study designs as compared to the cross-sectional approach taken herein. It may also be of interest that fewer regions exhibit reductions in surface area after values are normalized to the total brain surface area (%TBSA) (see Table 5). This includes the surface area of the white matter, the transverse temporal, anterior cingulate, lateral aspect of the superior temporal gyrus, the rostral anterior cingulate, and the medial orbital sulcus. A previous volumetric analysis on this same dataset reported many gray matter volumetric increases as a percentage of total brain volume and reported some regional white matter volumetric reductions [31]. Our findings of reduced white matter surface area agree with previous findings of reduced white matter volumes as a percentage of the estimated total intracranial volume [31] from an analysis on the same dataset.

### 4.5. Ventral Temporal Regions and Alzheimer’s Disease

A previous regional volumetric analysis of the brain in DS using the same dataset suggested that abnormalities in the entorhinal and perirhinal regions may be important in the etiology of Alzheimer’s disease (AD) in DS [31]. The current surface-based study identified specific structural abnormalities of the entorhinal and perirhinal regions in DS, which supports the potential connection of neurodevelopment of these regions in DS with AD [31]. The medial temporal lobe’s perirhinal cortex and entorhinal cortex provide neocortical and hippocampal connectivity, regions which are well implicated in memory [63]. More specifically, the perirhinal cortex and the parahippocampal cortex produce input to the hippocampus through entorhinal connections and obtain output through the entorhinal pathway from the hippocampus [64,65,66]. We observed increased surface area (%TBSA) in the perirhinal cortex, which is composed of Brodmann’s Areas (BA) 35 and 36 [67], as well as in the entorhinal cortex, which is composed of BA 28 [68], in DS. Animal-based research has implicated perirhinal cortical lesions in developmental impairments in recognition memory [69,70,71] and entorhinal cortical lesions were previously linked with memory deficits [72,73]. DS adults are at elevated risk of developing AD [16] and there is overlap between the neuropathology of AD and adults with DS over 40 years of age, including the presence of neurofibrillary tangles and senile plaques [74,75,76]. It was hypothesized that AD’s neuropathology was largely due to the overexpression of amyloid precursor protein (APP) as APP produces amyloid ß protein, the main component of senile plaques [77], and is located on chromosome 21. Decreased volumes of the entorhinal and perirhinal cortices have been reported in AD [68,78]. Despite known reductions in regional absolute volumes, it has been reported that the entorhinal cortex surface area did not present abnormally in AD [79]. Our findings also indicate no abnormal effect in the entorhinal cortex in DS in terms of raw/absolute surface area. However, when entorhinal surface area measurements were normalized by %TBSA, we observed large effect sizes in DS and an inversion of the common effect of reduced regional surface areas, to an increased regional surface area (%TBSA). Our findings imply that surface morphometry analyses may be particularly sensitive to abnormalities of the perirhinal and entorhinal regions, when assessed as %TBSA in DS. These findings help motivate future analyses in AD which focus on the potential for characterizing abnormal neurodevelopment with %TBSA measurements.

Leading observed abnormalities in DS included the perirhinal cortex and the entorhinal cortex (regions which are known to exhibit abnormalities in AD), findings that may be linked with the known increased prevalence of AD in DS. This is based on the understanding that these cortical regions are critical to memory function, along with the current findings and a previous analysis [31], and that abnormalities have been reported in these regions in both AD and DS. Although our participants were too young to typically start developing AD, we did observe early changes in the perirhinal cortex and in the entorhinal cortex, regions which have been linked with known memory deficits in AD. This has been reported in research results from fetal and neonatal brain MRI examinations of individuals with DS, which indicates that neurodevelopmental phenotypic abnormalities can be observed in fetal and neonatal brains [41,42]. Many of our results showed increased brain abnormalities as a function of age in the DS group relative to the neurotypicals. These findings combined with the current study offer evidence in support of the theory that developmental entorhinal and perirhinal abnormalities in DS progress with time/age, and may contribute to the memory problems associated with the development of AD in DS. Our findings imply that surface morphometry analyses may be particularly sensitive to abnormalities of the perirhinal and entorhinal regions and is worth extending to AD to enhance our understanding of the matter.

### 4.6. Strengths and Limitations

The strengths and limitations of our study design have been previously outlined [31,48,80,81]. A strength of the current study is the large sample size of neurotypical participants that were used to compare with the participants with DS. This provided a large baseline from which to assess statistical group differences. Another strength was that the dataset included several MRI examinations from the 0 to 5-year age range, a group that has been underrepresented in the literature. Strengths also include that this study performed a detailed anlaysis of surface morphometry measurements, which have the potential to assist in characterizing topology, which in the cerebral cortex presents as a highly convoluted two-dimensional sheet [45]. Traditional volumetric analyses [31] do not address the topological presentation of the brain’s cortex. Since cortical folding and sulci formation heavily affect the topology of the brain, and since they have been implicated as potentially important developmental characteristics of the brain [46,47], analyzing the DS population as part of a surface morphometry analysis may assist in better characterizing the presentation of the brain in DS with biomarker measurements that are more sensitive to cortical folding and sulci formation than traditional biomarkers, such as regional volumes. Limitations included the variability in imaging pulse sequence parameters due to variations in clinical pulse sequence use, and the modest sample size for DS participants, in particular the 10 to 15-year (n = 15) and 15 to 20-year (n = 9) age ranges. The small sample size at later ages and the lack of comprehensive longitudinal data on our patients make it very difficult to draw firm conclusions on aspects of brain development, and future work will need to confirm all the findings presented herein.

There were insufficient samples in the DS group to conduct reliable multivariable regression models to control or adjust for pulse sequence variability effects. This retrospective study (participants were referred for an MRI for clinical reasons and therefore may exhibit more extreme characteristics of DS), the lack of detailed patient interviews providing a complete comorbidity assessment, the lack of intelligence quotient (IQ) and other neurocognitive functional assessments of DS participants, and an imbalance between the numbers of participants in each group are additional limitations of this study. However, these findings offer an initial step towards further analyses in DS populations.

Bonferroni correction for the multiple comparisons problem was selected because it upholds the strictest standard in accepted methods for limiting the reporting of results that will not replicate in future analyses. However, it should be noted that such an analytic approach can result in the reporting of fewer results that achieve statistical significance (see bolded entries in Table 2, Table 3, Table 4 and Table 5 for statistically significant findings). While the Bonferroni correction results in fewer findings achieving statistical significance, it does help reduce the quantity of statistically significant findings that will not reproduce in future studies. Given that this study was based on real-world clinical data with substantial natural variability in both participant groups, this approach was selected to assist in helping to prevent the reporting of findings that will not be replicable. Finally, FreeSurfer was not optimized/validated for the youngest patients (0 to 8-months age range) causing considerable uncertainty in the reliability and reproducibility of findings from the youngest cohort. Fortunately, the youngest age group was not responsible for the primary findings of the current study. Research towards improving FreeSurfer’s performance in the youngest populations is a subject of ongoing research [82,83] and should be considered in future studies. Future studies should investigate whether the regional surface area abnormalities identified in the current study are related to functional deficits in DS, by analyzing detailed clinical data of each participant. Furthermore, researchers should consider incorporating other neuroimaging and analytic techniques (e.g., diffusion tensor imaging, functional MRI, and multivariate machine learning) to help improve our ability to characterize neurodevelopment associated with DS.

## 5. Conclusions

Brain surface morphometry analyses can potentially assist in providing a better understanding of structural brain differences, and may help characterize DS-specific neurodevelopment. Our retrospective surface morphometry study of MRI examinations of DS and neurotypical participants assessed surface curvature measurements, absolute surface area measurements, and surface areas as a percentage of total brain surface area (%TBSA). Results revealed broad reductions in surface area and abnormalities of surface curvature measurements across the brain in DS. After adjusting our regional surface area measurements as %TBSA, abnormally increased presentation in DS relative to neurotypical controls was observed in the left precentral, bilateral entorhinal, left parahippocampal, and bilateral perirhinal cortices, as well as Brodmann’s area 44 (left), and the right temporal pole. Findings suggest the presence of developmental abnormalities of regional %TBSA in DS that can be characterized from clinical MRI examinations.

## Figures and Tables

**Table 1:** Demographic information of participants.

Demographic Information and Comparative Statistics	Ages 0 to 5 Years	Ages 5 to 10 Years	Ages 10 to 15 Years	Ages 15 to 20 Years
DS—Mean age in years (SD)	2.12 (1.25)	7.33 (1.30)	13.78 (0.69)	16.35 (0.86)
Neurotypical—Mean age in years (SD)	2.59 (1.43)	7.63 (1.41)	12.41 (1.41)	16.70 (1.11)
DS—Age range in years	0.62–4.71	5.17–9.65	12.15–14.86	15.16–17.45
Neurotypical—Age range in years	0.00–4.99	5.02–9.98	10.04–14.99	15.01–19.95
DS—Ratio of males to females	17/9	12/10	10/5	7/2
Neurotypical—Ratio of males to females	71/68	124/137	115/177	80/194

DS: Down syndrome, SD: Standard deviation.

**Table 2:** Age-dependent analysis—Leading absolute surface area measurements (mm^2^) sorted by absolute effect size (Cohen’s d statistic) in descending order.

Region	Ages 0 to 5 Years L&R: d	Ages 5 to 10 Years L&R: d	Ages 10 to 15 Years L&R: d	Ages 15 to 20 Years L&R: d	MAX ABS (d)
Transverse temporal gyrus	L (−0.65) **R (−0.95)**	L (−0.99) R (−1.22)	**L (−1.84) R (−1.52)**	L (−1.18) R (−1.21)	1.84
Lateral aspect of the superior temporal gyrus	L (−0.63) **R (−0.97)**	**L (−1.44) R (−1.34)**	**L (−1.64) R (−1.83)**	L (−1.33) R (−1.44)	1.83
Transverse temporal sulcus	L (−0.67) R (−0.86)	**L (−1.01)** R (−0.96)	**L (−1.76)** R (−1.12)	L (−1.05) R (−0.78)	1.76
Inferior segment of the circular sulcus of the insula	L (−0.50) R (−0.83)	**L (−1.17) R (−1.45)**	**L (−1.67)** R (−1.28)	L (−1.09) **R (−1.40)**	1.67
Postcentral gyrus	**L (−0.94) R (−0.95)**	**L (−1.27)** R (−0.99)	**L (−1.62) R (−1.55)**	L (−0.62) R (−0.77)	1.62
Superior temporal gyrus	L (−0.63) **R (−0.94)**	**L (−1.53) R (−1.36)**	**L (−1.61) R (−1.60)**	L (−1.23) R (−1.23)	1.61
Anterior part of the cingulate gyrus and sulcus	L (−0.71) **R (−0.86)**	**L (−1.61) R (−1.34)**	**L (−1.60) R (−1.60)**	L (−1.44) R (−1.31)	1.61
Triangular part of the inferior frontal gyrus	L (−0.56) R (−0.45)	L (−0.72) R (−0.77)	**L (−1.58)** R (0.17)	L (−0.69) R (−0.41)	1.58
Lateral orbitofrontal gyrus	L (−0.66) R (−0.66)	**L (−1.55) R (−1.23)**	L (−1.37) R (−1.02)	**L (−1.46)** R (−0.92)	1.55
Rostral anterior cingulate	L (−0.56) R (−0.81)	**L (−1.54) R (−1.37)**	L (−1.17) R (−1.01)	L (−1.35) R (−0.77)	1.54
Supramarginal gyrus	L (−0.46) **R (−0.91)**	L (−0.82) R (−0.91)	L (−1.34) **R (−1.53)**	L (−0.80) R (−0.99)	1.53
Superior part of the precentral sulcus	L (−0.27) R (−0.36)	L (−0.21) R (−0.59)	**L (1.51)** R (0.26)	L (−0.07) R (−0.46)	1.51
Brodmann’s area V2	L (−0.62) R (−0.70)	**L (−1.02)** R (−0.95)	**L (−1.51)** R (−0.94)	L (−1.01) R (−0.86)	1.51
Brodmann’s area V1	L (−0.60) R (−0.69)	L (−0.98) R (−0.67)	**L (−1.51)** R (−0.93)	L (−0.88) R (−0.79)	1.51
White surface total area	L (−0.62) R (−0.72)	**L (−1.36) R (−1.31)**	**L (−1.49) R (−1.40)**	L (−1.02) R (−1.05)	1.49
Brodmann’s area 3b	**L (−0.88)** R (−0.84)	L (−0.94) R (−0.74)	**L (−1.48) R (−1.48)**	L (−0.85) R (−0.76)	1.48
Middle frontal gyrus	L (−0.59) R (−0.63)	**L (−1.18)** R (−1.01)	**L (−1.48)** R (−1.14)	L (−0.95) R (−0.50)	1.48
Cuneus gyrus	L (−0.54) R (−0.73)	L (−0.89) R (−0.84)	**L (−1.47)** R (−0.65)	L (−0.80) R (−1.17)	1.47
Pars triangularis	L (−0.50) R (−0.34)	L (−0.78) R (−0.74)	**L (−1.45)** R (−0.02)	L (−0.52) R (−0.30)	1.45
Orbital gyrus	L (−0.70) R (−0.49)	**L (−1.45) R (−1.12)**	L (−1.26) R (−0.83)	**L (−1.39)** R (−0.88)	1.45
Frontal pole	L (−0.59) R (−0.58)	L (−0.68) R (−0.52)	**L (−1.41) R (−1.44)**	L (−0.63) R (−0.38)	1.44
Transverse frontopolar gyrus and sulcus	L (−0.62) R (−0.54)	L (−0.73) R (−0.09)	L (−1.16) **R (−1.43)**	L (−0.58) R (−0.46)	1.43
Medial orbital sulcus	L (−0.55) R (−0.75)	**L (−1.29) R (−1.41)**	L (−1.30) R (−1.09)	**L (−1.38)** R (−1.34)	1.41
Brodmann’s area 45	L (−0.43) R (−0.41)	L (−0.88) R (−0.82)	**L (−1.40)** R (−0.29)	L (−0.59) R (−0.48)	1.40
Medial orbitofrontal	L (−0.49) R (−0.60)	**L (−1.22) R (−1.25)**	L (−1.18) **R (−1.40)**	L (−1.02) R (−1.18)	1.40
Rostral middle frontal	L (−0.37) R (−0.42)	L (−0.97) R (−0.72)	**L (−1.39)** R (−0.44)	L (−0.79) R (−0.41)	1.39
Pericalcarine	L (−0.62) R (−0.72)	L (−0.95) R (−0.68)	**L (−1.38)** R (−0.88)	L (−0.66) R (−0.86)	1.38
Middle-anterior cingulate gyrus and sulcus	L (−0.78) R (−0.79)	L (−1.00) **R (−1.09)**	L (−1.28) R (−1.22)	L (−1.11) **R (−1.36)**	1.36
Superior frontal gyrus	L (−0.71) R (−0.81)	**L (−1.23) R (−1.33)**	L (−1.20) R (−0.91)	L (−1.24) R (−0.93)	1.33
Planum temporale	L (−0.61) R (−0.65)	**L (−1.27)** R (−0.98)	L (−1.11) R (−0.63)	L (−1.02) R (−0.74)	1.27
Superior parietal	L (−0.79) **R (−0.89)**	**L (−1.24) R (−1.20)**	L (−0.96) R (−0.80)	L (−0.52) R (−0.73)	1.24
Brodmann’s area 2	L (−0.79) **R (−0.88)**	**L (−1.22)** R (−0.77)	L (−1.06) R (−1.34)	L (−0.22) R (−0.67)	1.22
Banks of the superior temporal sulcus	L (−0.58) R (−0.68)	**L (−1.19)** R (−0.91)	L (−1.27) R (−1.20)	L (−1.14) R (−0.91)	1.19
Insula	L (−0.41) R (−0.65)	L (−0.92) **R (−1.15)**	L (−0.91) R (−1.08)	L (−0.85) R (−1.25)	1.15
Lateral occipito-temporal sulcus	L (−0.36) R (−0.43)	L (−0.96) **R (−1.10)**	L (−0.64) R (−1.20)	L (−0.80) R (−1.21)	1.10
Gyrus rectus	L (−0.27) R (−0.52)	L (−0.81) **R (−1.09)**	L (−0.71) R (−1.23)	L (−0.75) R (−1.32)	1.09
Superior temporal sulcus	L (−0.40) R (−0.63)	**L (−1.09)** R (−0.98)	L (−0.58) R (−1.14)	L (−0.57) R (−0.94)	1.09
Brodmann’s area 6	L (−0.55) R (−0.69)	L (−0.88) **R (−1.08)**	L (0.08) R (−0.37)	L (−0.78) R (−0.82)	1.08
Superior segment of the circular sulcus of the insula	L (−0.62) R (−0.73)	L (−0.88) **R (−1.07)**	L (−1.06) R (−0.96)	L (−0.82) R (−0.65)	1.07
Parieto-occipital sulcus	L (−0.52) R (−0.66)	L (−0.75) **R (−1.05)**	L (−0.99) R (−0.39)	L (−0.37) R (−0.63)	1.05
Postcentral sulcus	L (−0.65) R (−0.73)	**L (−1.03)** R (−0.63)	L (−0.90) R (−1.09)	L (−0.07) R (−0.44)	1.03
H-shaped orbital sulcus	L (−0.59) R (−0.58)	**L (−1.02)** R (−0.74)	L (−0.76) R (−0.68)	L (−0.81) R (−0.35)	1.02
Brodmann’s area 1	**L (−1.00) R (−0.91)**	L (−1.00) R (−0.78)	L (−1.34) R (−1.34)	L (−0.37) R (−0.53)	1.00

Abbreviations/Symbols: R = right; L = Left; d = Cohen’s d statistic; MAX ABS = maximum absolute effect size (Cohen’s d). Bold entries indicate a statistically significant finding after multiple comparisons correction on at least one available FreeSurfer atlas.

**Table 3:** Age-dependent analysis—Leading surface curvature measurements sorted by absolute effect size (Cohen’s d statistic) in descending order.

Region	Ages 0 to 5 Years L&R: d	Ages 5 to 10 Years L&R: d	Ages 10 to 15 Years L&R: d	Ages 15 to 20 Years L&R: d	MAX ABS (d)
Entorhinal curvature index	L (0.45) R (−0.01)	L (0.29) R (0.38)	L (−0.07) R (−0.11)	**L (1.70) R (2.12)**	2.12
Transverse frontopolar gyri and sulci mean curvature	L (0.39) R (0.04)	L (0.15) R (−0.20)	**L (−2.03)** R (−1.31)	L (−0.34) R (0.02)	2.03
Entorhinal mean curvature	L (0.53) R (−0.08)	L (0.80) R (0.47)	L (0.12) R (−0.13)	L (1.31) **R (2.00)**	2.00
Superior and transverse occipital sulci Gaussian curvature	L (−0.16) R (−0.15)	L (−0.10) R (−0.03)	L (−0.04) R (−0.07)	L (0.93) **R (1.94)**	1.94
Whole brain intrinsic curvature index (positive)	**L (−0.94) R (−0.96)**	**L (−1.17) R (−1.09)**	**L (−1.88) R (−1.81)**	L (−0.28) R (−0.16)	1.88
Whole brain intrinsic curvature index (negative)	**L (−0.94) R (−0.96)**	**L (−1.17) R (−1.09)**	**L (−1.88) R (−1.81)**	L (−0.28) R (−0.16)	1.88
Inferior temporal sulcus curvature index	L (−0.03) R (0.41)	L (0.36) R (0.04)	L (0.30) R (−0.29)	**L (1.83) R (1.58)**	1.83
Superior and transverse occipital sulci folding index	L (−0.31) R (−0.01)	L (−0.15) R (−0.16)	L (−0.11) R (−0.47)	L (0.14) **R (1.82)**	1.82
Parahippocampal gyrus mean curvature	L (0.26) R (0.16)	L (0.87) R (0.37)	L (0.00) R (−0.27)	L (1.15) **R (1.78)**	1.78
Frontal pole folding index	L (−0.16) R (0.30)	L (−0.18) R (−0.23)	L (−0.30) R (−0.12)	L (−0.08) **R (1.71)**	1.71
Middle temporal gyrus gaussian curvature	L (−0.11) R (0.13)	L (−0.20) R (−0.24)	L (−0.17) R (−0.28)	L (0.05) **R (1.71)**	1.71
Middle occipital gyrus Gaussian curvature	L (−0.13) R (−013)	L (0.04) R (−0.09)	L (−0.23) R (−0.24)	**L (1.68)** R (0.27)	1.68
Entorhinal cortex Gaussian curvature	L (0.11) R (0.22)	L (0.04) R (0.38)	L (−0.06) R (−0.11)	**L (1.67)** R (1.09)	1.67
Brodmann’s area 44 folding index	L (0.31) R (0.30)	L (−0.20) R (−0.14)	L (−0.07) R (−0.21)	L (−0.02) **R (1.66)**	1.66
Perirhinal cortex mean curvature	L (0.25) R (0.17)	L (0.60) R (0.53)	L (−0.16) R (0.12)	L (1.25) **R (1.66)**	1.66
Brodmann’s area 44 Gaussian curvature	L (0.31) R (0.45)	L (−0.07) R (0.03)	L (−0.05) R (−0.03)	L (0.13) **R (1.65)**	1.65
Rostral middle frontal mean curvature	L (−0.04) R (−0.19)	L (−0.25) R (−0.40)	**L (−1.52) R (−1.61)**	L (−0.01) R (0.01)	1.61
Anterior transverse collateral sulcus folding index	L (0.64) R (−0.09)	L (−0.19) R (−0.09)	L (−0.25) R (−0.16)	**L (1.61)** R (−0.05)	1.61
Inferior temporal cortex mean curvature	L (0.10) R (0.17)	L (0.94) R (1.03)	L (−0.38) R (0.05)	L (0.90) **R (1.61)**	1.61
Medial orbitofrontal cortex folding index	L (−0.12) R (−0.24)	L (−0.18) R (−0.07)	L (−0.25) R (−0.29)	L (−0.13) **R (1.60)**	1.60
Perirhinal cortex curvature index	L (0.03) R (0.48)	L (−0.05) R (0.27)	L (0.06) R (−0.04)	L (1.26) **R (1.59)**	1.59
Gyrus rectus curvature index	L (−0.14) R (−0.20)	L (0.03) R (0.40)	L (−0.39) R (0.12)	L (−0.08) **R (1.58)**	1.58
Opercular part of the inferior frontal gyrus Gaussian curvature	L (0.07) R (0.44)	L (−0.07) R (−0.03)	L (−0.33) R (−0.09)	L (0.12) **R (1.57)**	1.57
Pars opercularis Gaussian curvature	L (0.38) R (−0.08)	L (−0.08) R (−0.11)	L (−0.15) R (−0.21)	L (0.21) **R (1.57)**	1.57
Pericallosal sulcus Gaussian curvature	L (−0.13) R (−0.24)	L (0.54) R (0.59)	L (0.07) **R (1.53)**	L (−0.08) R (0.16)	1.53
Anterior part of the cingulate gyrus and sulcus folding index	L (−0.10) R (−0.06)	L (−0.26) R (−0.26)	L (−0.69) R (−0.65)	L (1.25) **R (1.53)**	1.53
Inferior frontal sulcus folding index	L (−0.20) R (−0.28)	L (−0.39) R (−0.20)	L (−0.42) R (−0.17)	L (−0.07) **R (1.51)**	1.51
Lateral orbitofrontal folding index	L (−0.17) R (0.23)	L (0.48) R (−0.32)	L (−0.44) R (−0.04)	L (−0.03) **R (1.51)**	1.51
Pars orbitalis mean curvature	L (−0.08) R (0.30)	L (0.31) R (0.09)	L (−0.49) **R (−1.51)**	L (0.05) R (0.12)	1.51
Medial orbital sulcus mean curvature	L (0.17) R (0.54)	L (0.85) R (0.91)	L (0.14) R (0.63)	L (0.72) **R (1.51)**	1.51
Fusiform folding index	L (−0.05) R (0.09)	L (0.17) R (0.12)	L (−0.21) R (−0.30)	**L (1.50)** R (−0.05)	1.50
Whole hemisphere folding index	**L (−1.00) R (−0.95)**	**L (−1.10) R (−1.17)**	**L (−1.41) R (−1.48)**	L (−0.58) R (−0.55)	1.48
Entorhinal folding index	L (−0.11) R (0.56)	L (−0.01) R (−0.06)	L (−0.08) R (−0.12)	L (0.43) **R (1.48)**	1.48
Brodmann’s area 45 Gaussian curvature	L (−0.06) R (−0.13)	L (−0.07) R (0.03)	L (−0.10) R (−0.08)	**L (1.47)** R (1.32)	1.47
Medial occipito-temporal and lingual sulci curvature index	L (−0.11) R (−0.23)	L (0.48) R (−0.02)	L (0.19) R (−0.18)	**L (1.47)** R (0.71)	1.47
Perirhinal cortex Gaussian curvature	L (−0.11) R (0.44)	L (0.20) R (−0.03)	L (−0.07) R (−0.11)	**L (1.47)** R (1.16)	1.47
Brodman’s area 45 mean curvature	L (0.23) R (−0.08)	L (0.45) R (−0.07)	L (−1.19) **R (−1.44)**	L (0.60) R (0.56)	1.44
Posterior cingulate Gaussian curvature	L (0.27) R (−0.25)	L (0.54) R (−0.18)	L (−0.16) **R (1.44)**	L (0.02) R (1.44)	1.44
Frontal pole Gaussian curvature	L (−0.03) R (0.05)	L (−0.26) R (−0.22)	L (−0.36) R (−0.10)	L (0.25) **R (1.44)**	1.44
Triangular part of the inferior frontal gyrus curvature index	L (−0.25) R (−0.18)	L (−0.10) R (−0.14)	L (−0.50) R (−0.11)	L (0.89) **R (1.44)**	1.44
Anterior part of the cingulate gyrus and sulcus curvature index	L (−0.14) R (−0.15)	L (−0.56) R (−0.42)	L (−1.25) **R (−1.41)**	L (0.11) R (−0.02)	1.41
Medial occipito-temporal and lingual sulci mean curvature	L (0.25) R (−0.08)	**L (1.41)** R (0.88)	L (0.32) R (0.20)	**L (1.41)** R (0.75)	1.41
Orbital gyrus mean curvature	L (−0.21) R (0.20)	L (−0.43) R (−0.36)	**L (−1.39)** R (−1.11)	L (0.13) R (0.21)	1.39
Lingual gyrus mean curvature	L (0.20) R (0.22)	**L (1.35)** R (0.92)	L (0.09) R (−0.84)	L (0.99) R (0.69)	1.35
Cuneus gyrus mean curvature	L (−0.05) R (0.09)	**L (1.12)** R (0.63)	L (0.20) R (−0.56)	L (0.74) R (1.03)	1.12
Medial occipito-temporal and lingual sulci Gaussian curvature	L (−0.03) R (−0.12)	**L (1.11)** R (0.03)	L (−0.04) R (0.01)	L (1.10) R (0.54)	1.11
V1 mean curvature	L (0.02) R (0.19)	**L (1.11)** R (0.46)	L (−0.25) R (−0.31)	L (0.69) R (0.62)	1.11
V2 mean curvature	L (0.05) R (0.22)	**L (1.05)** R (0.57)	L (0.19) R (−1.22)	L (0.85) R (0.69)	1.05

Abbreviations/Symbols: R = right; L = Left; d = Cohen’s d statistic; MAX ABS = maximum absolute effect size (Cohen’s d). Bold entries indicate a statistically significant finding after multiple comparisons correction on at least one available FreeSurfer atlas.

**Table 4:** Age-dependent analysis—Leading surface area measurements as a %TBSA sorted by effect size (Cohen’s d statistic) (positive effects—larger in DS) in descending order.

Region	Ages 0 to 5 Years L&R: d	Ages 5 to 10 Years L&R: d	Ages 10 to 15 Years L&R: d	Ages 15 to 20 Years L&R: d	MAX ABS (d)
Superior part of the precentral sulcus	L (0.29) R (0.29)	L (0.32) R (−0.09)	**L (2.22)** R (0.88)	L (0.39) R (−0.11)	2.22
Precentral cortex	L (0.47) R (0.32)	L (1.00) R (0.44)	**L (1.84)** R (0.46)	L (0.97) R (0.37)	1.84
Entorhinal cortex	L (0.54) R (0.35)	L (0.66) R (0.72)	**L (1.73) R (1.35)**	L (1.19) R (0.40)	1.73
Parahippocampal cortex	L (0.36) R (−0.02)	L (0.13) R (0.59)	**L (1.61)** R (0.06)	L (0.99) R (0.24)	1.61
Perirhinal cortex	L (0.44) R (0.23)	L (0.37) R (0.49)	**L (1.49) R (1.58)**	L (0.92) R (0.93)	1.58
Temporal pole	L (−0.08) R (−0.07)	L (0.49) **R (1.13)**	L (0.65) R (0.33)	L (0.20) R (0.84)	1.13
Brodmann’s area 44	**L (0.96)** R (0.76)	L (0.92) R (0.79)	L (0.81) R (0.42)	L (1.07) R (0.53)	0.96
Inferior part of the precentral sulcus	**L (0.95)** R (0.75)	L (0.78) R (0.77)	L (0.97) R (1.13)	L (0.88) R (0.51)	0.95

Abbreviations/Symbols: R = right; L = Left; d = Cohen’s d statistic; MAX ABS = maximum absolute effect size (Cohen’s d). Bold entries indicate a statistically significant finding after multiple comparisons correction on at least one available FreeSurfer atlas.

**Table 5:** Age-dependent analysis for leading surface area measurements as a percentage of total brain surface area (%TBSA) sorted by effect size (Cohen’s d statistic) (negative effects—smaller in DS).

Region	Ages 0 to 5 Years L&R: d	Ages 5 to 10 Years L&R: d	Ages 10 to 15 Years L&R: d	Ages 15 to 20 Years L&R: d	MAX ABS (d)
White matter total surface area	L (0.04) R (−0.70)	L (−0.83) R (−0.32)	**L (−1.52)** R (−1.17)	L (−0.34) R (−0.66)	1.52
Transverse temporal sulcus	L (−0.33) R (−0.58)	L (−0.62) R (−0.47)	**L (−1.51)** R (−0.86)	L (−0.75) R (−0.44)	1.51
Transverse temporal gyrus	L (−0.19) R (−0.68)	L (−0.41) R (−0.47)	**L (−1.48)** R (−1.15)	L (−0.82) R (−0.88)	1.48
Anterior cingulate gyrus and sulcus	L (−0.43) R (−0.75)	**L (−1.37)** R (−0.89)	L (−1.32) R (−1.35)	**L (−1.47)** R (−1.18)	1.47
Lateral aspect of the superior temporal gyrus	L (−0.11) R (−0.51)	L (−0.86) R (−0.63)	L (−0.98) **R (−1.38)**	L (−0.94) R (−1.12)	1.38
Rostral anterior cingulate	L (−0.39) R (−0.70)	**L (−1.33) R (−1.10)**	L (−0.77) R (−0.61)	L (−1.38) R (−0.45)	1.33
Medial orbital sulcus	L (−0.23) R (−0.55)	L (−0.93) **R (−1.08)**	L (−0.94) R (−0.68)	L (−1.30) R (−1.21)	1.08

Abbreviations/Symbols: R = right; L = Left; d = Cohen’s d statistic; MAX ABS = maximum absolute effect size (Cohen’s d). Bold entries indicate a statistically significant finding after multiple comparisons correction on at least one available FreeSurfer atlas.

## Data Availability

The data used in this analysis are private clinical data.

## Supplementary Materials

The following supporting information can be downloaded at: https://www.mdpi.com/article/10.3390/biology13080575/s1. Figure S1: Black and white scatter plots of the leading findings presented in Table 4 and Table 5, representing surface area abnormalities identified in DS as a percentage of total brain surface area (%TBSA). Green samples represent neurotypical participants, red samples represent DS participants. X represents a male, O a female.
