# Supplementary material for: Structural Magnetic Resonance Imaging-Based Surface Morphometry Analysis of Pediatric Down Syndrome
Source: Biology (Basel). 2024 Jul 30;13(8):575. doi: 10.3390/biology13080575 (PMC11351698; doi:10.3390/biology13080575)
Supplement: Supplementary file 1 [file biology-13-00575-s001.zip › biology-3045276-supplementary.pdf]

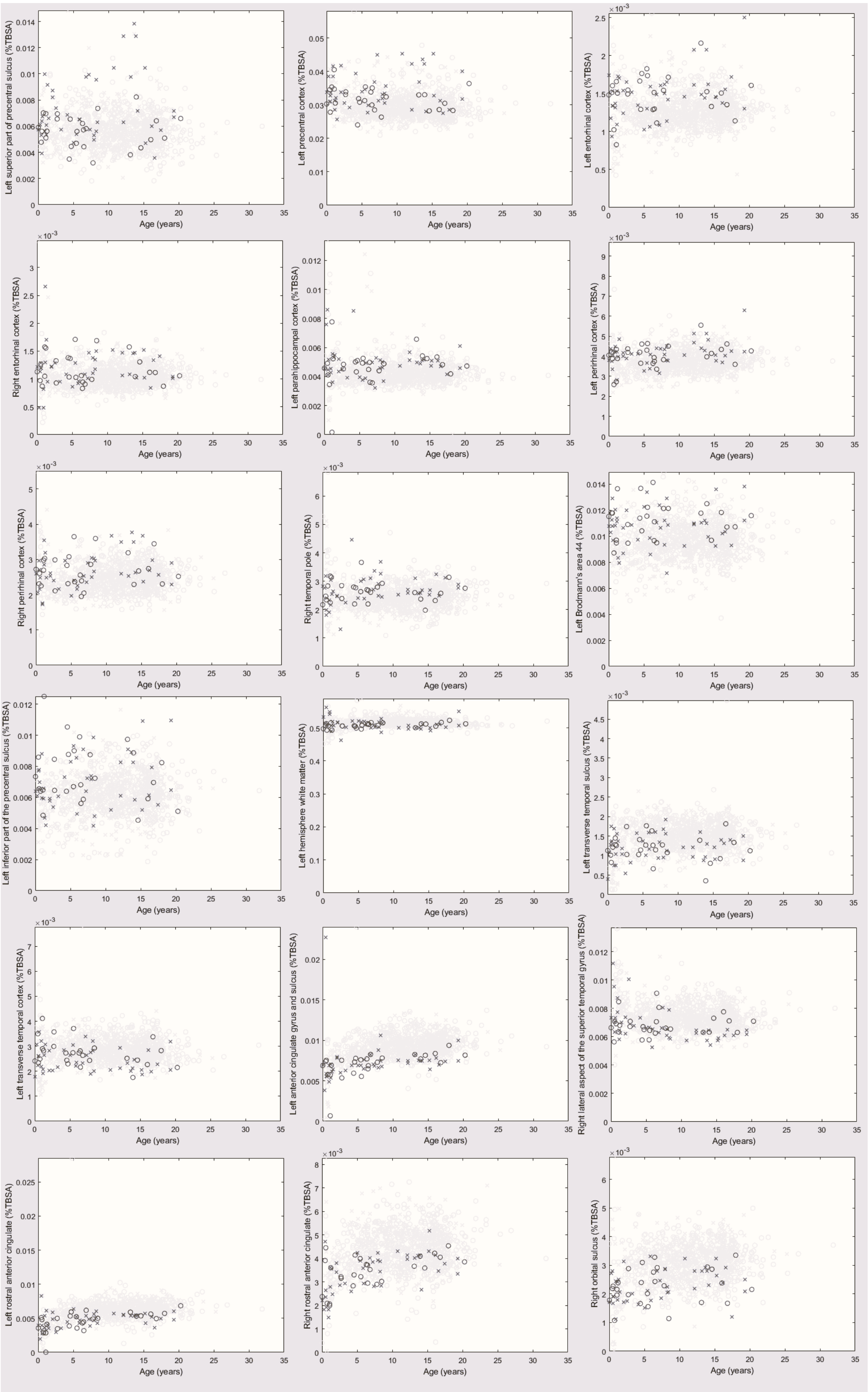

Left superior part of precentral sulcus (%TBSA)
Left precentral cortex (%TBSA)
Left entorhinal cortex (%TBSA)
Right entorhinal cortex (%TBSA)
Left parahippocampal cortex (%TBSA)
Left perirhinal cortex (%TBSA)
Right perirhinal cortex (%TBSA)
Right temporal pole (%TBSA)
Left Brodmann's area 44 (%TBSA)
Left inferior part of the precentral sulcus (%TBSA)
Left hemisphere white matter (%TBSA)
Left transverse temporal sulcus (%TBSA)
Left transverse temporal cortex (%TBSA)
Left anterior cingulate gyrus and sulcus (%TBSA)
Right lateral aspect of the superior temporal gyrus (%TBSA)
Left rostral anterior cingulate (%TBSA)
Right rostral anterior cingulate (%TBSA)
Right orbital sulcus (%TBSA)
Age (years)

Figure S1. Black and white scatter plots of the leading findings presented in Tables 4 and 5, representing surface area abnormalities identified in DS as a percentage of total brain surface area (%TBSA). X represents a male, O a female.
